# Perioperative dexmedetomidine plus esketamine for postoperative neurocognitive outcomes: a systematic review and meta-analysis

**DOI:** 10.3389/fmed.2026.1909826

**Published:** 2026-08-20

**Authors:** Zhi-yong Wang, Wen-jun Yuan, Jun Pu, Yan-bin Yin

**Affiliations:** Department of Anesthesia, Chengdu Xinhua Hospital Affiliated to North Sichuan Medical College, Chengdu, China

**Keywords:** dexmedetomidine, esketamine, meta-analysis, neuroprotection, postoperative cognitive dysfunction, postoperative delirium

## Abstract

**Background:**

Perioperative neurocognitive disorders (PND), including postoperative cognitive dysfunction (POCD) and postoperative delirium (POD), are common complications after surgery and are associated with poor clinical outcomes. This systematic review and meta-analysis aimed to evaluate the effects of perioperative combined administration of dexmedetomidine and esketamine on postoperative neurocognitive function and perioperative recovery outcomes.

**Methods:**

PubMed, Embase, Web of Science, Cochrane Library, CNKI, and the Chinese Medical Journal Full-text Database were systematically searched from database inception to April 2026. Randomized controlled trials and prospective cohort studies evaluating perioperative dexmedetomidine combined with esketamine were included. The primary outcomes were postoperative neuronal injury biomarkers and cognitive function scores. Secondary outcomes included postoperative delirium, postoperative nausea and vomiting (PONV), and postoperative pain scores. Meta-analysis was performed using RevMan and Stata software, and evidence quality was assessed using the GRADE approach.

**Results:**

Eight studies involving 1,118 patients were included, comprising seven randomized controlled trials and one prospective cohort study. Compared with controls, combined dexmedetomidine and esketamine was associated with lower postoperative 24-h serum neuron-specific enolase (NSE) levels (SMD = −0.95, 95% CI: −1.16 to −0.73, *I*^2^ = 18%) and S-100β levels (SMD = −1.17, 95% CI: −1.39 to −0.95, *I*^2^ = 0%). The combined intervention was also associated with higher MMSE scores (SMD = 0.84, 95% CI: 0.05 to 1.63, *I*^2^ = 91%) and MoCA scores (SMD = 0.49, 95% CI: 0.20 to 0.79, *I*^2^ = 19%). In addition, the risks of POD (RR = 0.45, 95% CI: 0.29 to 0.70, *I*^2^ = 0%) and PONV (RR = 0.50, 95% CI: 0.35 to 0.72, *I*^2^ = 0%) were lower, and early postoperative pain scores were lower in the combined intervention group (SMD = −0.51, 95% CI: −0.87 to −0.14, *I*^2^ = 69%). Sensitivity analyses demonstrated stable results. According to the GRADE assessment, the quality of evidence ranged from moderate to low, and the 24-h cognitive test scores should be interpreted as short-term postoperative assessments.

**Conclusion:**

The findings suggest that perioperative combined administration of dexmedetomidine and esketamine may be associated with attenuated postoperative neuronal injury, improved early cognitive function, and reduced risks of POD, PONV, and pain. Further high-quality multicenter randomized controlled trials are warranted to confirm these preliminary findings.

## Highlights

- Perioperative combined administration of dexmedetomidine and esketamine may be associated with lower postoperative serum levels of neuronal injury biomarkers NSE and S-100β, suggesting a potential neuroprotective effect.
- The combination may be associated with improved early postoperative cognitive function, as reflected by higher MMSE and MoCA scores, and was associated with a lower incidence of postoperative delirium (POD).
- The combination may decrease the risk of postoperative nausea and vomiting (PONV) and may improve early postoperative pain control, potentially through opioid-sparing mechanisms.
- The current evidence is of moderate to low quality; therefore, high quality, large scale multicenter randomized controlled trials are needed to confirm these findings and define optimal dosing regimens.

## Introduction

Postoperative cognitive dysfunction (POCD) and postoperative delirium (POD) are common central nervous system complications following surgery under general anesthesia, particularly among elderly patients and those undergoing major surgery (1). Perioperative neurocognitive disorders (PND), as a unified terminology, encompass two major clinical entities: POCD is characterized by persistent impairment in memory, attention, and executive function, whereas acute, fluctuating disturbances in attention and consciousness characterize POD (2). These complications not only prolong hospitalization and substantially increase healthcare costs, but are also closely associated with increased postoperative all-cause mortality, long-term cognitive decline, and elevated risk of dementia (3). With the accelerating aging of the population and the continuous increase in surgical procedures, postoperative neurocognitive disorders pose major challenges to patient health, healthcare professionals, and public health systems. Effective prevention of postoperative cognitive impairment has therefore become a central issue in perioperative management (4).

The concept of enhanced recovery after surgery (ERAS) promotes postoperative recovery by systematically reducing surgical stress responses and protecting organ function through multimodal interventions (5). Within this framework, exploration of pharmacological combinations with both neuroprotective and sedative–analgesic properties is of substantial clinical importance. Esketamine, an N-methyl-D-aspartate (NMDA) receptor antagonist, possesses rapid antidepressant and analgesic effects. Although it may improve postoperative pain and mood disorders, its net effect on postoperative cognitive function remains controversial. A recent high-quality randomized controlled trial demonstrated that perioperative low-dose esketamine significantly alleviated postoperative depressive symptoms but failed to reduce the incidence of POD or POCD (6). Dexmedetomidine, a highly selective α2-adrenergic receptor agonist, exerts arousable sedative, anti-inflammatory, and neuroprotective effects. Clinical studies have confirmed that dexmedetomidine significantly reduces the incidence of POCD in elderly patients undergoing radical gastrectomy and suppresses the release of peripheral inflammatory cytokines (7).

Monotherapy has limitations: esketamine's cognitive effects are uncertain, and dexmedetomidine, though effective, needs optimization. Thus, recent studies have focused on their combined, potentially synergistic effects. Existing evidence suggests that dexmedetomidine combined with esketamine may exert significant synergistic neuroprotective effects in elderly patients undergoing abdominal surgery. This combination not only suppresses intraoperative hemodynamic fluctuations and maintains an appropriate depth of anesthesia to preserve cerebral perfusion, but also synergistically inhibits the release of peripheral and central inflammatory cytokines, thereby reducing the incidence of POCD at various postoperative time points (8). Furthermore, a previous meta-analysis demonstrated that dexmedetomidine also exerts protective effects against POD and POCD during regional anesthesia (9).

Nevertheless, systematic evaluations regarding the effects of this combination on postoperative cognitive function remain limited. Existing studies exhibit heterogeneity in surgical type, timing of administration, and dosing regimens, which restricts the standardized clinical application of this combined strategy.

Therefore, the present study conducted a systematic review and meta-analysis to comprehensively synthesize current available clinical evidence and evaluate the effects of perioperative combined administration of esketamine and dexmedetomidine on postoperative cognitive function. In addition, the effects of this combination on neuronal injury biomarkers, pain, postoperative nausea and vomiting, and other perioperative outcomes were further explored to provide preliminary evidence for optimizing perioperative neuroprotective strategies.

## Methods

### Literature review and search strategy

This systematic review and meta-analysis were conducted in accordance with the PRISMA 2020 guidelines. The study protocol was prospectively registered on the PROSPERO platform (CRD420261397656). Ethical approval was not required because this study was based exclusively on previously published data.

### Inclusion and exclusion criteria

The inclusion and exclusion criteria were established according to the PICOS framework.

#### Inclusion criteria

1. The study design primarily consisted of randomized controlled trials (RCTs). Given the limited number of studies in this field, high-quality prospective cohort studies were also considered when the number of RCTs was insufficient for meta-analysis.
2. Participants were adult patients aged ≥18 years undergoing elective surgery under general anesthesia.
3. The intervention group received perioperative (preoperative, intraoperative, or postoperative) combined administration of esketamine and dexmedetomidine, whereas the control group received placebo (e.g., normal saline), conventional sedative/analgesic regimens, or single-drug interventions (esketamine alone or dexmedetomidine alone).
4. At least one perioperative neurocognitive-related outcome was reported, including: (1) biomarkers of neuronal injury (NSE, S-100β); (2) postoperative cognitive assessment scores (MMSE, MoCA); (3) the incidence of postoperative delirium (POD) or postoperative cognitive dysfunction (POCD); and (4) other perioperative recovery outcomes (PONV, VAS pain scores).
5. Full texts and complete data were available.

#### Exclusion criteria

1. Non-clinical studies, including reviews, Meta-analyses, case reports, animal experiments, and conference abstracts.
2. Studies involving non-surgical patients or patients not receiving general anesthesia.
3. Studies that failed to report or provide extractable outcome data.
4. Studies with unclear combined medication regimens or concomitant use of other interventions potentially affecting cognitive function (e.g., therapeutic hypothermia).
5. Duplicate publications, for which only the most complete or most recent study was included.

### Literature search strategy

CNKI, Chinese Medical Journal Full-text Database, Cochrane Library, Embase, PubMed, and Web of Science were systematically searched from database inception to April 2026. Both Medical Subject Headings and free-text terms were used. Chinese search terms included “艾司氯胺酮,” “S-氯胺酮,” “右美托咪定,” “术后认知功能障碍,” “术后谵妄,” “认知障 碍,” and “神经认知障碍.” English search terms included “esketamine,” “S-ketamine,” “dexmedetomidine,” “postoperative cognitive dysfunction,” “postoperative delirium,” “cognitive impairment,” and “neurocognitive disorder.” The search strategy was designed to capture primary clinical studies; terms such as “meta-analysis” or “systematic review” were not used as main search terms to avoid retrieving secondary publications. The detailed PubMed search strategy is presented in Supplementary Table S1.

### Literature screening, data extraction, and quality assessment

Study selection and data extraction were independently performed by two investigators. Disagreements were resolved through discussion or adjudication by a third investigator when necessary. Data extraction was conducted using a standardized Excel form, including first author, publication year, country, study type, sample size, patient age, surgical type, anesthetic regimen, dosage, timing, and route of administration of esketamine and dexmedetomidine, control measures, and outcome indicators (NSE, S-100β, MMSE, MoCA, POD, PONV, and VAS scores). For missing data, corresponding authors were contacted via email. If no response was received within 14 days, the data were treated as unavailable.

### Risk-of-bias assessment

The methodological quality of randomized controlled trials was evaluated using the Cochrane RoB 2.0 tool across five domains: randomization process, deviations from intended interventions, missing outcome data, outcome measurement, and selective reporting (10). Prospective cohort studies were assessed using the Newcastle–Ottawa Scale (NOS) (11).

### Quality of evidence assessment

The quality of evidence for each outcome was evaluated using the GRADE (Grading of Recommendations Assessment, Development and Evaluation) approach and categorized as high, moderate, low, or very low quality (12).

### Statistical analysis

Meta-analysis and forest plots were performed using Review Manager 5.3, whereas Stata 12.0 was used for heterogeneity exploration, sensitivity analysis, and publication bias assessment. Continuous variables were expressed as mean difference (MD) or standardized mean difference (SMD) with 95% confidence intervals (CI), whereas dichotomous variables were expressed as risk ratio (RR) with 95%CI. Between-study heterogeneity was assessed using the χ^2^ test and *I*^2^ statistic. A fixed-effects model was applied when *I*^2^ ≤ 50% and *P* > 0.10; otherwise, a random-effects model was used. Considering the potential clinical heterogeneity among studies in terms of surgical type, anesthetic regimen, and drug administration strategies, random-effects models were preferentially used when necessary. When substantial heterogeneity was identified, subgroup analyses based on surgical type, drug regimen, and anesthesia method were planned. Sensitivity analyses were performed by sequentially excluding individual studies to evaluate the robustness of pooled results. Funnel plots and Egger's regression tests were planned when at least 10 studies were included for a specific outcome. A two-sided *P* < 0.05 was considered statistically significant.

## Results

### Literature screening process and results

**Figure 1:**
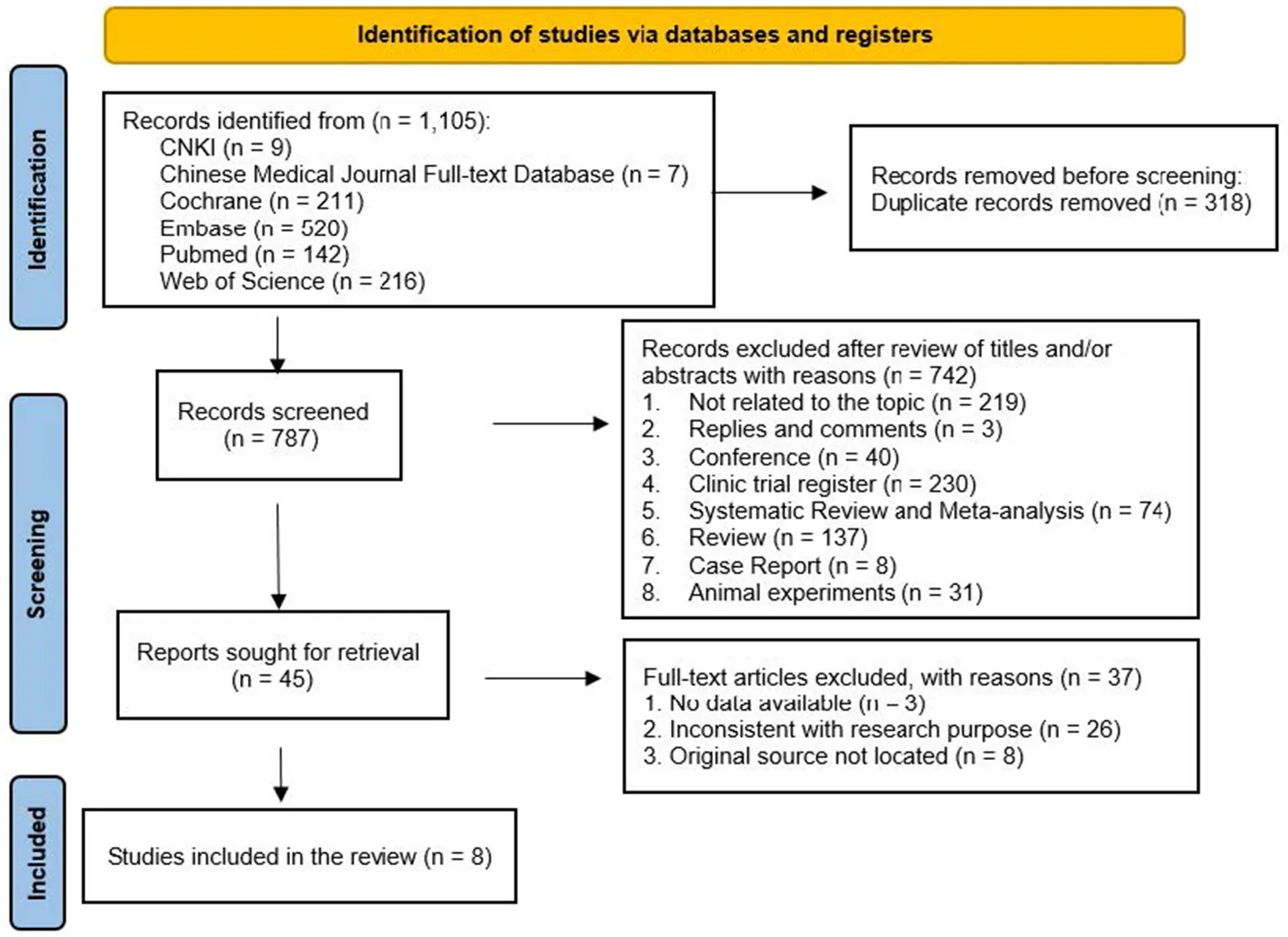
PRISMA flow diagram of literature search and study selection.

A total of 1,105 relevant studies were initially identified (CNKI: 9; Chinese Medical Journal Full-text Database: 7; Cochrane Library: 211; Embase: 520; PubMed: 142; Web of Science: 216). After duplicate removal, 787 studies remained. Screening of titles and abstracts excluded 742 studies, including 219 irrelevant studies, 3 comments or replies, 40 conference abstracts, 230 clinical trial registrations, 74 systematic reviews/Meta-analyses, 137 reviews, 8 case reports, and 31 animal studies. Subsequently, 45 articles underwent full-text assessment. During full-text screening, 37 studies were excluded because of unavailable data (*n* = 3), inconsistency with the study objective (*n* = 26), or inaccessible original articles (*n* = 8). Ultimately, eight studies were included in the qualitative and quantitative synthesis (meta-analysis), comprising seven randomized controlled trials and one prospective cohort study. The literature screening flowchart is shown in Figure 1.

### Characteristics and quality assessment of included studies

All included studies were conducted in China. Surgical procedures included laparoscopic hysterectomy (13, 14), thoracoscopic radical resection (15), lumbar surgery (16), hip surgery (17), laparoscopic cholecystectomy (18), spinal surgery (19), and radical mastectomy (20). Among the eight studies, two incorporated regional nerve blocks (erector spinae plane block or transversus abdominis plane block), whereas the remaining six employed general anesthesia alone. Patient age ranged from 44 to 75 years, and the majority of participants were classified as American Society of Anesthesiologists (ASA) physical status II–III, although some studies included ASA I or III patients. Overall, sex distribution varied according to surgical type: gynecological and breast surgeries included only female patients, whereas other surgical categories exhibited relatively balanced sex distributions. Baseline characteristics, including age, sex, ASA classification, body mass index (BMI), and operative duration, were generally comparable between the intervention and control groups (Table 1).

**Table 1:** Characteristics of included studies and baseline patient data.

References	Country	Surgery type	Study design	Regional block	Sample size (E/C)	Age (years, E/C)	Sex (M/F, E/C)	ASA physical status	BMI (kg/m^2^, E/C)	Surgery duration (min, E/C)
Mao and Wang (13)	China	Laparoscopic hysterectomy	RCT	None	52/52	44.25 ± 7.54/44.52 ± 5.92	0/52 vs. 0/52	I–II	24.18 ± 1.87/23.78 ± 1.59	85.50 ± 16.69/85.90 ± 16.58
Zhang et al. (15)	China	Thoracoscopic radical resection	RCT	ESPB	82/81	70.35 ± 3.77/70.64 ± 3.77	38/44 vs. 42/39	II–III	23.19 ± 3.54/22.60 ± 2.86	50.64 ± 9.81/55.59 ± 7.54
Li et al. (14)	China	Laparoscopic hysterectomy	Prospective cohort study	None	59/55	65.2 ± 5.4/64.8 ± 5.6	0/59 vs. 0/55	II–III	26.8 ± 3.5/27.1 ± 4.0	136.76 ± 26.59/141.76 ± 26.64
Tao et al. (16)	China	Lumbar surgery	RCT	None	53/53	71.9 ± 7.7/72.2 ± 6.2	20/33 vs. 20/33	II–III	23.8 ± 3.3/23.6 ± 3.2	152.42 ± 53.34/138.71 ± 47.24
Ye et al. (17)	China	Hip surgery	RCT	None	60/61	72.41 ± 10.62/73.70 ± 12.15	24/36 vs. 26/35	II–III	21.70 ± 3.03/22.64 ± 3.79	NR
Li et al. (18)	China	Laparoscopic cholecystectomy	RCT	TAP	45/45	70 ± 5/71 ± 4	21/24 vs. 23/22	II–III	21 ± 2/21.4 ± 1.5	59 ± 18/61 ± 15
Wang et al. (19)	China	Spinal surgery	RCT	None	45/44	71.25 ± 2.99/71.36 ± 3.01	24/21 vs. 23/22	I–III	21.11 ± 0.61/21.15 ± 0.65	NR
Zhou et al. (20)	China	Breast cancer radical surgery	RCT	None	40/40	66.0 ± 3.5/66.0 ± 3.7	0/40 vs. 0/40	I–III	NR	94.1 ± 14.3/98.9 ± 16.1

E, experimental group; C, control group; RCT, randomized controlled trial; ASA, American Society of Anesthesiologists; BMI, body mass index; ESPB, erector spinae plane block; TAP, transversus abdominis plane block; NR, not reported. Data are presented as mean ± standard deviation or n.

Detailed anesthetic and perioperative management protocols are summarized in Table 2, including dexmedetomidine and esketamine dosing regimens (loading and maintenance doses), timing of administration, infusion strategies, and duration of infusion. All studies used general anesthesia (combined intravenous–inhalational anesthesia or total intravenous anesthesia). The intervention groups received perioperative combined administration of dexmedetomidine and esketamine, primarily during anesthesia induction, although dosing regimens and maintenance protocols varied. Control groups received opioids alone, dexmedetomidine alone, esketamine alone, or conventional patient-controlled intravenous analgesia. Some studies adopted opioid-free anesthesia strategies (e.g., Li et al. (14), Ye et al. (17), Li et al. (18)). In most studies, anesthetic depth was adjusted according to bispectral index (BIS 40–60) and hemodynamic parameters.

**Table 2:** Perioperative anesthesia protocols and intraoperative management.

References	Surgery type	Anesthesia type	Regional block	Dexmedetomidine	Esketamine	Timing of administration	Infusion regimen	Duration	Postoperative analgesia
Mao and Wang (13)	Laparoscopic hysterectomy	Intravenous-inhalational	None	0.3 μg/kg (single bolus)	0.5 mg/kg (single bolus)	Before induction (preoperative)	Single bolus via pump	~10 min (single dose)	PCIA: sufentanil 100 μg + dezocine 15 mg + ketorolac 120 mg
Zhang et al. (15)	Thoracoscopic radical resection	Intravenous-inhalational + ESPB	ESPB (0.25–0.5% ropivacaine 15–20 mL)	Loading: 0.5 μg/kg Maintenance: 0.4 μg/kg/h	Loading: 0.25 mg/kg Maintenance: 0.125 mg/kg/h	Before induction + intraoperative	Loading bolus + continuous infusion	From induction until 30 min before end of surgery	PCIA: sufentanil 0.5–1 μg/kg + dezocine 0.3–0.5 mg/kg
Li et al. (14) (Prospective^a^)	Laparoscopic hysterectomy	OFA: TIVA (opioid-free) Control: Intravenous-inhalational	None	Bolus: 0.5 μg/kg Maintenance: 0.3 μg/kg/h	Bolus: 0.25 mg/kg Maintenance: 0.20 mg/kg/h	Before induction + intraoperative	Bolus then continuous infusion	Stopped 20 min before skin closure	Lidocaine 1.5 mg/kg/h + ketorolac 30 mg (opioid-free)
Tao et al. (16)	Lumbar spinal surgery	Intravenous-inhalational	None (local ropivacaine 40 mg infiltration)	0.4 μg/kg (single dose before induction)	0.5 mg/kg (single dose after intubation)	Before induction (dex)/After intubation (esket)	Single bolus only	Single dose (no intraoperative maintenance)	PCIA: flurbiprofen 150 mg + sufentanil 2 μg/kg + dexamethasone 10 mg
Ye et al. (17)	Hip surgery	OFA: TIVA (opioid-free) Control: Intravenous-inhalational	Preoperative fascia iliaca block	Loading: 0.7 μg/kg Maintenance: continued infusion	Maintenance: 0.25 mg/kg/h	Before induction + intraoperative	Loading bolus + continuous infusion	Intraoperative continuous infusion	Tramadol 30 mg (rescue analgesia)
Li et al. (18)	Laparoscopic cholecystectomy	OFA: Intravenous-inhalational + TAP Control: Intravenous-inhalational	TAP block (0.25% ropivacaine 20 mL)	Loading: 1 μg/kg Maintenance: 0.5 μg/kg/h	Loading: 0.5 mg/kg Maintenance: 0.25 mg/kg/h	Before induction + intraoperative	Loading bolus + continuous infusion	Continuous infusion during surgery	Ketorolac 30 mg + metoclopramide 10 mg (IV)
Wang et al. (19)	Spinal surgery	TIVA	None	Loading: 1 μg/kg (10 min) Maintenance: 0.5 μg/kg/h	3 mg/kg IM (single loading for both groups)	Intraoperative (dex: infusion) (esket: single IM dose)	Dex: loading then infusion Esket: single IM dose only	Dex: continuous infusion Esket: single dose	Flurbiprofen 50 mg/12 h PCIA: sufentanil 100 μg + tropisetron 10 mg
Zhou et al. (20)	Radical mastectomy	TIVA	None	2 μg/kg (via PCIA)	2 mg/kg (via PCIA)	End of surgery → postoperative (PCIA)	PCIA continuous infusion	48 h postoperatively (PCIA)	PCIA: dex 2 μg/kg + esket 2 mg/kg + tropisetron 5 mg in 100 mL

^a^Li et al. (14) is a prospective cohort study; all other studies are RCTs. BIS, bispectral index; ESPB, erector spinae plane block; IM, intramuscular; OFA, opioid-free anesthesia; PCIA, patient-controlled intravenous analgesia; TAP, transversus abdominis plane block; TIVA, total intravenous anesthesia.

The reported outcome indicators are presented in Table 3, which details for each study the specific cognitive function tests used, POD and POCD events separately, neuronal injury biomarkers, pain scores, PONV events, safety outcomes, assessment timing, assessment tools, and follow-up duration. Primary outcomes included postoperative 24-h serum NSE and S-100β protein levels (3 studies), as well as postoperative 24-h MMSE and MoCA scores (5 studies). Secondary outcomes included the incidence of POD (reported in 7 studies, with 5 studies using standardized POD definitions and included in the pooled analysis), incidence of PONV (7 studies), and postoperative VAS pain scores at different time points (4 studies reporting 2-h VAS scores).

**Table 3:** Outcomes included in quantitative synthesis.

References	Cognitive function	POD	POCD	Neuronal biomarkers	Pain scores	PONV	Safety outcomes (bradycardia, hypotension, hallucination, etc.)	Assessment timing	Assessment tools	Follow-up duration
Mao and Wang (13)	MMSE	–	18/52 vs. 29/52	NSE (24 h) S-100β (24 h)	–	Yes	Hypotension: 3.8% vs. 7.7% Bradycardia: 17.3% vs. 15.4% Nausea/vomiting: 7.7% vs. 7.7% Skin itch: 0% vs. 1.9%	MMSE: preop, D1, D3, D7 NSE/S-100β: preop, end surg, 6 h, 24 h	POCD: MMSE decline ≥3 pts from baseline	7 days
Zhang et al. (15)	QoR-15	Within 7 days	–	NSE (T0–T5) S-100β (T0–T5)	NRS	Yes	Nausea/vomiting: 14.6% vs. 28.4% Delirium: 14.6% vs. 30.9%	NSE/S-100β: 6 timepoints (T0–T5) POD: daily within 7 days	POD: 3D-CAM	7 days (POD) 48 h (other)
Li et al. (14)	MoCA	Postop (CAM-ICU)	–	–	NRS	Yes	Delirium: 5.1% vs. 9.1% Sedation requiring intervention: 3.4% vs. 10.9% Serious AEs: 1.7% vs. 3.6%	MoCA: preop, D1, D3 Delirium: postop	POD: CAM-ICU MoCA score	3 days
Tao et al. (16)	MMSE, MoCA	–	6/53 vs. 13/53	NSE (24 h) S-100β (24 h)	VAS 2 h: 1.64 ± 0.76 vs. 2.64 ± 2.28 (24 h/48 h NR)	Yes	Nausea/vomiting: 9.4% (ED) vs. 13.2% (D) (Other outcomes not systematically reported)	MMSE/MoCA: preop, D1, D3 NSE/S-100β: preop, 24 h	POCD: ≥1 SD decline from preop MMSE/MoCA	3 days
Ye et al. (17)	–	Within 48 h	–	–	VAS 2 h: 2.82 ± 1.13 vs. 2.92 ± 1.36 (24 h/48 h NR)	Yes (composite)	Composite AEs: 35.0% (OFA) vs. 62.3% (control) Hypoxemia: lower in OFA Hypotension: lower in OFA Bradycardia: lower in OFA Delirium: comparable	VAS: 2, 24, 48 h Composite AEs: within 48 h	POD: CAM	48 h
Li et al. (18)^a^	–	Postop	–	–	–	Yes	Bradycardia: 11% (OFA) vs. 22% (GA) Hypotension: lower in OFA Nausea: 29% vs. 16% Vomiting: 18% vs. 4% Delirium: 4% vs. 18%	Delirium: postop	POD: not clearly specified (monitored as AE)	24 h (PACU)
Wang et al. (19)	MMSE	–	–	–	VAS (2, 4, 6, 8 h)	Yes	Total AEs: 6.67% vs. 22.73% Sedation, respiratory depression, nausea/vomiting (Bradycardia/hypotension not systematically reported)	MMSE: D1, D3, D7 VAS: 2, 4, 6, 8 h postop	N/A (no POD/POCD assessed)	7 days
Zhou (20)	–	Within 48 h	–	–	VAS (2, 4, 8, 24, 48 h)	Yes	Nightmare: lower in Dex+Esket vs. Esket alone Delirium: 2% vs. 20% vs. 2% Hallucinations: comparable Dizziness, nausea: lower in Dex+Esket Hypotension: comparable	VAS: 2, 4, 8, 24, 48 h Delirium: within 48 h	POD: not clearly specified (recorded as AE)	48 h

^a^Li et al. (14) is a prospective cohort study; all other studies are RCTs. “–” indicates the outcome was not reported in that study. **Note on safety outcomes:** Most studies did not systematically report all prespecified safety outcomes (e.g., hypertension, emergence agitation, delayed recovery). The absence of standardized safety reporting is a limitation of the current evidence base, as acknowledged in the Discussion. **Follow-up duration:** reflects the maximum protocol-defined observation period for cognitive or safety outcomes in each study. Short follow-up durations (< 7 days) limit the ability to diagnose persistent postoperative neurocognitive disorder per DSM-5 criteria. 3D-CAM, 3-Min diagnostic interview for CAM-defined delirium; AE, adverse event; CAM, confusion assessment method; CAM-ICU, confusion assessment method for the intensive care unit; ED, esketamine + dexmedetomidine group; GA, general anesthesia; IM, intramuscular; MMSE, mini-mental state examination; MoCA, montreal cognitive assessment; N/A, not applicable; NRS, numeric rating scale; NR, not reported; NSE, neuron-specific enolase; OFA, opioid-free anesthesia; POCD, postoperative cognitive dysfunction; POD, postoperative delirium; PONV, postoperative nausea and vomiting; QoR-15, 15-item quality of recovery; SD, standard deviation; VAS, visual analog scale.

**Figure 2:**
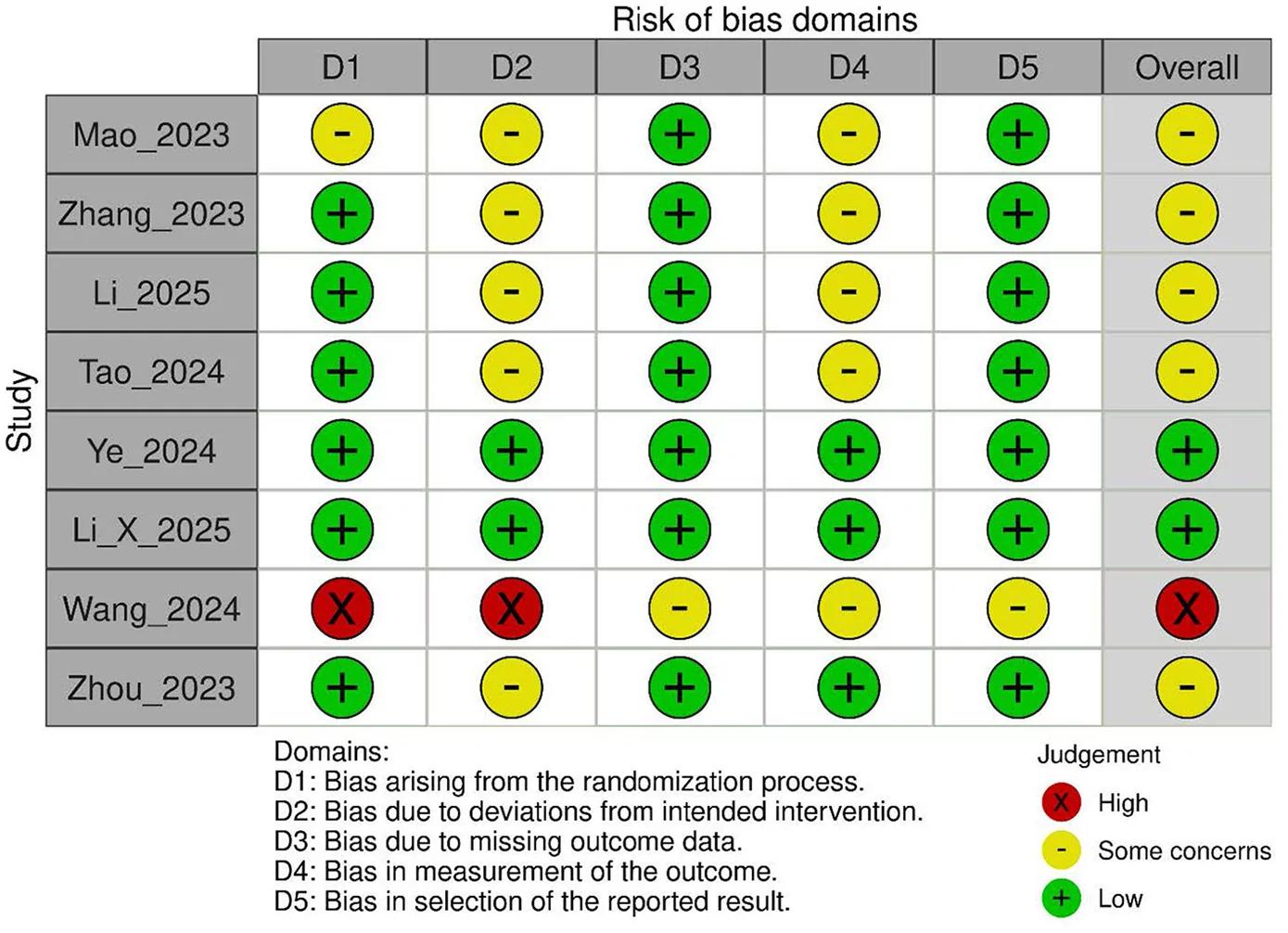
Risk of bias assessment of the included studies using the Cochrane ROB 2.0 tool.

The methodological quality of the seven included randomized controlled trials was assessed using the Cochrane ROB 2.0 tool. As shown in Figure 2, all seven studies were judged as having a low risk of bias in random sequence generation. Regarding allocation concealment, three studies were considered low risk, and four were unclear risk. For blinding of participants and personnel, six studies were assessed as low risk and one as unclear risk. All studies were judged as low risk in outcome assessment, blinding, completeness of outcome data, and selective reporting. In addition, the included prospective cohort study (Li et al.) was evaluated using the NOS. This study controlled confounding bias through propensity score matching (PSM), implemented blinded outcome assessment, and achieved a score of 8/9, indicating high methodological quality.

### Meta-analysis results

#### Primary outcomes

##### Postoperative 24-h neuronal injury biomarkers (NSE and S-100β)

**Figure 3:**
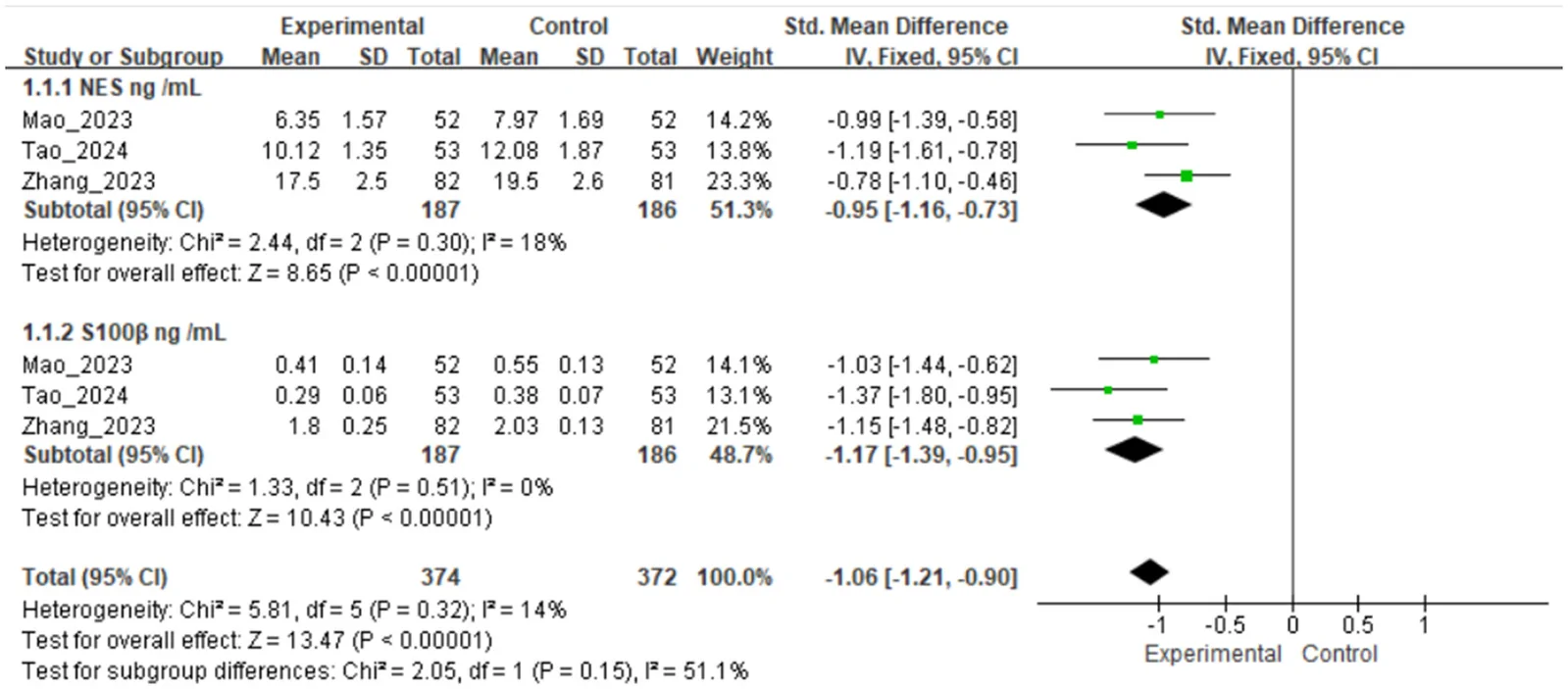
Forest plot of the effects of esketamine combined with dexmedetomidine on postoperative NSE and S-100β levels.

Three studies involving 373 patients (187 in the intervention group and 186 in the control group) reported postoperative 24-h serum NSE and S-100β levels. A fixed-effects model was used because heterogeneity was low (*I*^2^ < 50%). Meta-analysis demonstrated that, compared with the control group, esketamine combined with dexmedetomidine was associated with lower NSE levels (SMD = −0.95, 95%CI: −1.16 to −0.73, *P* < 0.001, *I*^2^ = 18%) and S-100β levels (SMD = −1.17, 95%CI: −1.39 to −0.95, *P* < 0.001, *I*^2^ = 0%). The overall pooled analysis also demonstrated a significant advantage of the combined intervention over the control group (SMD = −1.06, 95%CI: −1.22 to −0.91, *P* < 0.001, *I*^2^ = 14%; Figure 3).

##### Postoperative 24-h cognitive function scores (MMSE and MoCA)

**Figure 4:**
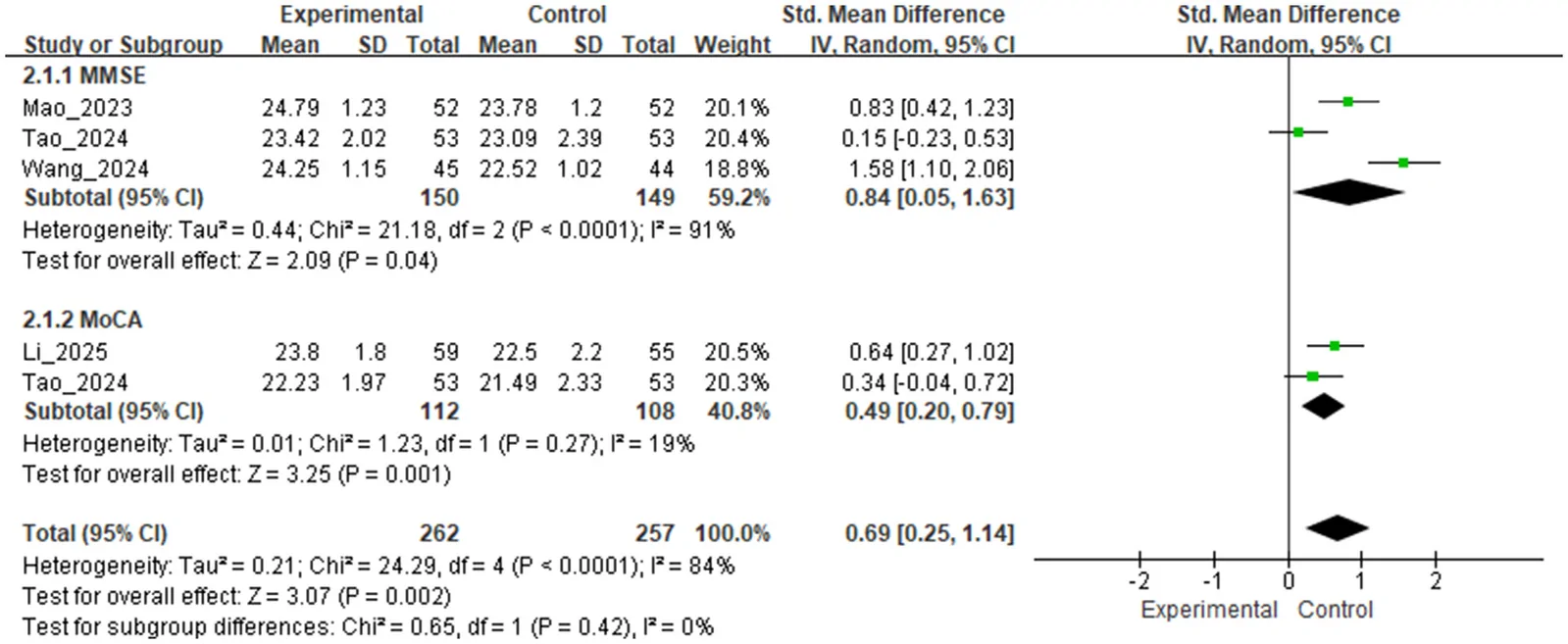
Forest plot of postoperative cognitive function outcomes following perioperative esketamine combined with dexmedetomidine.

Five studies reported postoperative 24-h cognitive function scores. Among them, three studies involving 299 patients reported MMSE scores, whereas two studies involving 220 patients reported MoCA scores. Because substantial heterogeneity was observed (*I*^2^ > 50%), a random-effects model was applied. The results demonstrated that the combined intervention significantly improved MMSE scores (SMD = 0.84, 95%CI: 0.05 to 1.63, *P* = 0.04, *I*^2^ = 91%) and MoCA scores (SMD = 0.49, 95%CI: 0.20 to 0.79, *P* = 0.001, *I*^2^ = 19%). Subgroup difference testing revealed no significant difference in effect sizes between the two cognitive scales (*P* = 0.42, *I*^2^ = 0%). Overall pooled analysis also indicated that the combined intervention were associated with higher postoperative 24-h overall cognitive function scores (SMD = 0.69, 95%CI: 0.25 to 1.14, *P* = 0.002, *I*^2^ = 84%). Despite substantial heterogeneity in the MMSE analysis (*I*^2^ = 91%), no single study drove the pooled effect in sensitivity analyses, indicating the overall estimate is robust (Figure 4) These 24-h cognitive test scores should be interpreted as early postoperative cognitive test scores rather than definitive evidence of neurocognitive protection.

#### Secondary outcomes

##### Postoperative delirium (POD)

Five studies involving 568 patients (286 in the intervention group and 282 in the control group) were included. It should be noted that Mao and Wang (13) reported POCD rather than POD. Given the differences in clinical definitions and assessment tools between these two conditions, this study was not included in the pooled POD analysis. A fixed-effects model was applied (*I*^2^ = 0%). The results demonstrated that esketamine combined with dexmedetomidine was associated with a lower incidence of POD compared with the control group (RR = 0.45, 95%CI: 0.29 to 0.70, *P* < 0.01), with low heterogeneity (*I*^2^ = 0%), indicating stable findings.

##### Postoperative nausea and vomiting (PONV)

Seven studies involving 746 patients (376 in the intervention group and 370 in the control group) were included. Fixed-effects pooled analysis (*I*^2^ = 0%) demonstrated that the incidence of PONV was lower in the combined intervention group than in the control group (RR = 0.50, 95%CI: 0.35 to 0.72, *P* < 0.01).

##### Postoperative 2-h VAS pain scores

**Figure 5:**
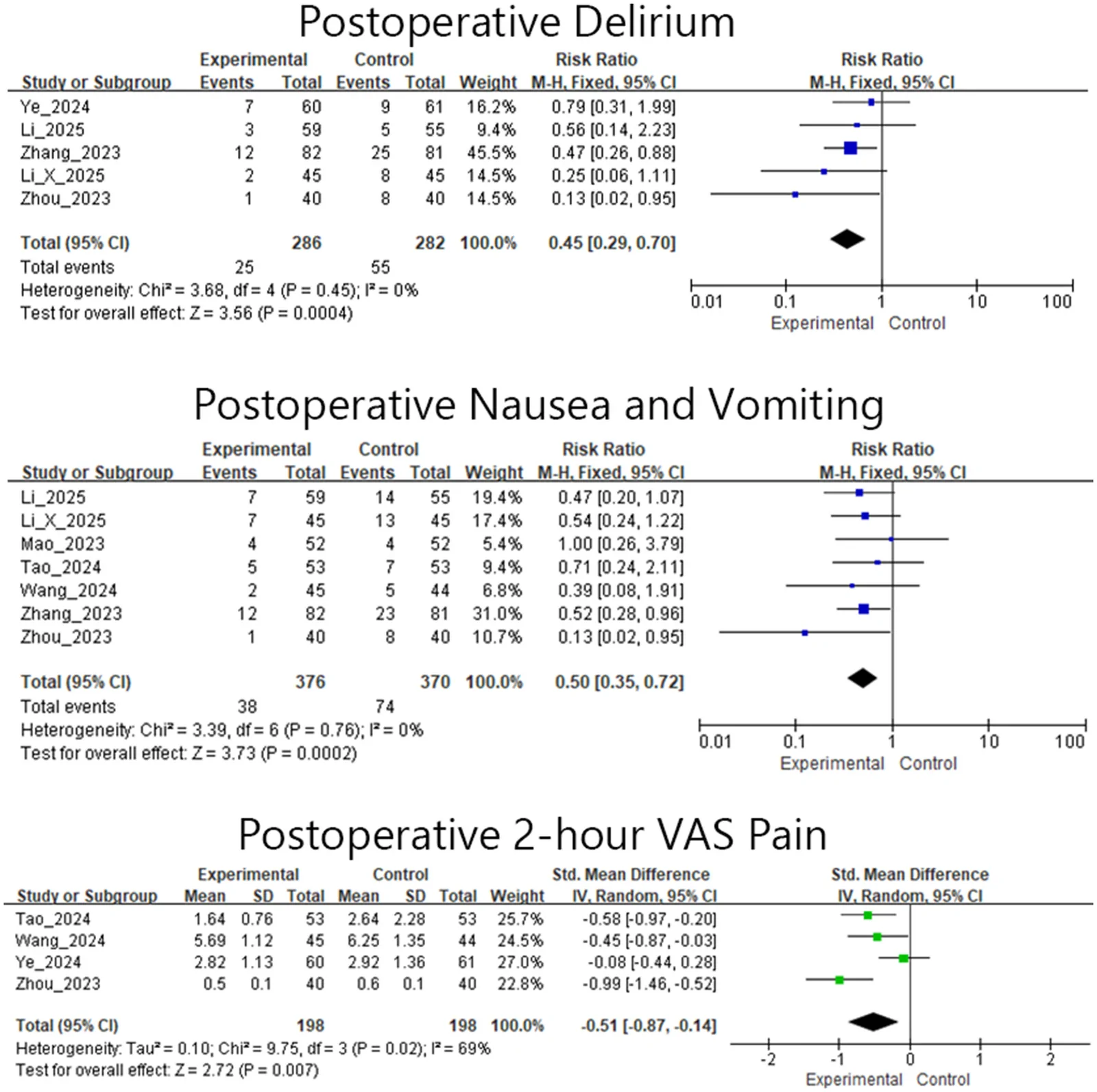
Forest plot of postoperative delirium, postoperative nausea and vomiting, and postoperative pain outcomes.

Four studies involving 396 patients (198 patients in each group) were included. Because moderate heterogeneity was observed (*I*^2^ = 69%), a random-effects model was applied. The results demonstrated that the combined intervention associated with lower postoperative 2-h VAS pain scores (SMD = −0.51, 95%CI: −0.87 to −0.14, *P* = 0.007). The observed heterogeneity may have resulted from differences in pain assessment timing, surgical type, or baseline analgesic regimens. Sensitivity analysis did not identify any single study exerting a decisive influence on the pooled effect (Figure 5).

### Sensitivity analysis

Sensitivity analyses using sequential study exclusion (leave-one-out approach) were performed to evaluate the robustness of pooled outcomes. The results for each outcome are presented in Supplementary Figure S1.

For postoperative 24-h cognitive function scores (MMSE and MoCA), leave-one-out sensitivity analysis demonstrated that exclusion of any single study yielded pooled SMD estimates with 95% confidence intervals consistently below the null line, confirming stable results.

For postoperative 24-h cognitive function scores (MMSE and MoCA), leave-one-out analysis showed that exclusion of Wang et al. (19) resulted in the largest change in the pooled MMSE estimate, while exclusion of Mao and Wang (13) reduced the *I*^2^ value from 91% to 88%, indicating that this study partially contributed to the observed heterogeneity. Exclusion of any single study did not cause the 95% CI to cross the null line.

For POD, PONV, and postoperative 2-h VAS pain scores, leave-one-out analyses consistently demonstrated that exclusion of any single study did not reverse the direction or significance of the pooled effects, confirming the robustness of the findings.

In addition, a sensitivity analysis excluding the only non-randomized study (Li et al. (14), a prospective cohort study) was performed. The results remained consistent with the main analyses, indicating that the conclusions were not disproportionately influenced by the inclusion of this observational study (Supplementary Figure S2).

Sensitivity analyses for QoR-15 were not performed because this outcome was reported by only one study (Zhang et al. (15)) and could not be pooled.

Although a formal publication bias assessment was not performed, fewer than 10 studies were included for each outcome. According to the PRISMA 2020 guidelines, funnel plots and Egger's tests have insufficient statistical power under such conditions and were therefore not conducted. Publication bias cannot be excluded, particularly given that all included studies are single-country trials with predominantly positive findings.

### Evidence quality assessment

The quality of evidence for primary and secondary outcomes was assessed using the GRADE system, and the results are summarized in Table 4. For primary outcomes, the quality of evidence for postoperative 24-h NSE and S-100β levels was rated as moderate because of imprecision. The quality of evidence for postoperative 24-h MMSE scores was rated as low owing to inconsistency (*I*^2^ = 91%) and imprecision. The quality of evidence for postoperative 24-h MoCA scores was rated as moderate because of imprecision. Regarding secondary outcomes, the quality of evidence for POD and PONV was rated as moderate, with no serious concerns regarding inconsistency, indirectness, or risk of bias. The quality of evidence for postoperative 2-h VAS pain scores was rated as low, primarily because of moderate heterogeneity (*I*^2^ = 69%). Overall, the estimated effects of the combined intervention on neuronal injury biomarkers, POD, and PONV demonstrated moderate certainty, whereas the certainty of evidence for postoperative cognitive function (MMSE) and early postoperative pain scores was low, warranting cautious interpretation (Table 4).

**Table 4:** Summary of findings and GRADE certainty assessment for esketamine combined with dexmedetomidine in surgical patients.

Outcomes	No. of studies (participants)	Risk of bias	Inconsistency	Indirectness	Imprecision	Publication bias	Effect estimate (SMD/RR, 95% CI)	Certainty of evidence (GRADE)
Primary outcomes								
NSE level at 24 h postoperatively	3 (373)	Not serious^a^	Not serious (*I*^2^ = 18%)	Not serious	Serious^b^	Suspected^c^	SMD = −0.95 (−1.16 to −0.73)	⨁⨁⨁◯ Moderate
S-100β level at 24 h postoperatively	3 (373)	Not serious^a^	Not serious (*I*^2^ = 0%)	Not serious	Serious^b^	Suspected^c^	SMD = −1.17 (−1.39 to −0.95)	⨁⨁⨁◯ Moderate
MMSE score at 24 h postoperatively	3 (299)	Not serious^a^	Serious^d^ (*I*^2^ = 91%)	Not serious	Serious^e^	Suspected^c^	SMD = 0.84 (0.05 to 1.63)	⨁⨁◯◯ Low
MoCA score at 24 h postoperatively	2 (220)	Not serious^a^	Not serious (*I*^2^ = 19%)	Not serious	Serious^f^	Suspected^c^	SMD = 0.49 (0.20 to 0.79)	⨁⨁⨁◯ Moderate
Secondary outcomes								
Postoperative delirium (POD)	5 (568)	Not serious^a^	Not serious (*I*^2^ = 0%)	Not serious	Not serious	Suspected^c^	RR = 0.45 (0.29 to 0.70)	⨁⨁⨁◯ Moderate
Postoperative nausea and vomiting (PONV)	7 (746)	Not serious^a^	Not serious (*I*^2^ = 0%)	Not serious	Not serious	Suspected^c^	RR = 0.50 (0.35 to 0.72)	⨁⨁⨁◯ Moderate
VAS pain score at 2 h postoperatively	4 (396)	Not serious^a^	Serious^g^ (*I*^2^ = 69%)	Not serious	Not serious	Suspected^c^	SMD = −0.51 (−0.87 to −0.14)	⨁⨁◯◯ Low

^a^Most included studies were randomized controlled trials with an overall low risk of bias according to the ROB 2.0 assessment tool.

^b^The total sample size was limited and did not meet the optimal information size (OIS), resulting in downgraded certainty for imprecision.

^c^Formal assessment of publication bias was not feasible because fewer than 10 studies were included for each outcome. Given that all included studies were small single-country trials with predominantly positive findings, publication bias cannot be excluded.

^d^Considerable heterogeneity was observed across studies (*I*^2^ = 91%).

^e^The confidence interval was relatively wide and approached the line of no effect, indicating uncertainty in the pooled estimate.

^f^Only two studies with a limited sample size were available for analysis.

^g^Moderate heterogeneity may be attributable to differences in surgical type, perioperative analgesic protocols, and timing of pain assessment.

## Discussion

This systematic review and meta-analysis found that perioperative dexmedetomidine combined with esketamine was associated with reduced postoperative NSE and S-100β levels, improved MMSE and MoCA scores at 24 h, and lower incidences of POD, PONV, and early postoperative pain. These associations are encouraging, but they should be interpreted as preliminary rather than definitive, given the moderate-to-low certainty of the underlying evidence.

To our knowledge, this is the first meta-analysis to evaluate the combined regimen specifically for neurocognitive outcomes. Earlier meta-analyses have addressed each agent separately: dexmedetomidine was shown to reduce POD in a 2018 meta-analysis, though with safety concerns (21); perioperative S-ketamine provided analgesia and opioid-sparing effects, yet its cognitive benefit remained inconclusive (22). The present findings raise the possibility that the two drugs together might offer broader perioperative advantages than either alone, potentially through complementary actions on neuroinflammation, nociceptive processing, and sympathetic tone. However, this mechanistic inference remains speculative and requires direct experimental validation.

The potential mechanisms underlying these findings are likely multifactorial. Esketamine attenuates glutamate-mediated excitotoxicity primarily through N-methyl-D-aspartate (NMDA) receptor antagonism, thereby reducing neuronal calcium overload and apoptosis. Emerging evidence further suggests that esketamine may exert anti-inflammatory and antioxidative effects through suppression of NF-κB-mediated signaling pathways (23). Recent clinical studies have also demonstrated anti-inflammatory and immunomodulatory effects of esketamine in surgical patients (24). In contrast, dexmedetomidine exerts sedative and neuroprotective effects mainly through activation of central α2-adrenergic receptors, thereby reducing sympathetic activation, stabilizing hemodynamics, and attenuating neuroinflammation (25). Experimental evidence further suggests that dexmedetomidine may regulate postoperative cognitive dysfunction through autophagy-related pathways (26). The complementary pharmacological actions of these two agents may therefore contribute to preservation of neuronal integrity and blood–brain barrier stability, as reflected by the reductions in NSE and S-100β observed in the present analysis. Experimental studies have also suggested that the combination of dexmedetomidine and esketamine may exert synergistic anti-inflammatory and neuroprotective effects (27).

The lower PONV and pain scores observed with the combination warrant cautious interpretation. Both dexmedetomidine and esketamine have opioid-sparing properties, and reduced opioid exposure likely contributes to these benefits (28). However, this explanation is complicated by the heterogeneity of control groups: some studies used single-drug comparators, some used opioid-based anesthesia, and others used conventional care. Moreover, three of the included studies adopted opioid-free anesthesia protocols, and four incorporated regional analgesic techniques (erector spinae plane block, fascia iliaca block, transversus abdominis plane block, or local infiltration). These co-interventions independently reduce PONV and pain, making it difficult to isolate the specific contribution of the drug combination itself. The pooled estimates may thus reflect the cumulative effect of multimodal analgesic strategies rather than a pure pharmacological signal.

Substantial heterogeneity was observed in MMSE-related outcomes. Removal of Mao et al. (2023) notably reduced heterogeneity, likely because the study also used regional anesthesia, which exerts opioid-sparing and anti-inflammatory effects. Differences in surgical invasiveness, anesthetic protocols, drug timing, and cognitive assessment methods may also have contributed. Notably, the heterogeneity did not reverse the direction of effect, and sensitivity analyses supported the robustness of the overall estimate.

A notable gap in the included studies is the near-absence of safety reporting. None of the trials systematically reported bradycardia, hypotension, hypertension, hallucinations, emergence agitation, or delayed recovery associated with the combination. Given that both drugs have well-documented hemodynamic and psychotomimetic side-effect profiles, this omission limits the clinical applicability of the findings. Future studies should prioritize systematic safety assessment alongside efficacy outcomes.

Several limitations should be acknowledged. First, the total sample size remains modest, and all eight studies originated from China, which restricts generalizability to other populations and healthcare settings. Perioperative practices and genetic polymorphisms affecting drug metabolism may differ across ethnic groups. Second, the variability in surgical procedures, anesthetic techniques, and control regimens introduced unavoidable clinical heterogeneity. Third, follow-up was short-term in most studies, so the effect on persistent postoperative neurocognitive disorders (beyond the immediate postoperative period) remains unknown. Fourth, although the overall methodological quality was acceptable, GRADE assessment downgraded the certainty of evidence for several outcomes because of inconsistency and imprecision. Fifth, publication bias cannot be excluded, especially given the single-country origin of all included studies and the predominance of positive results, although the small number of studies per outcome precluded formal assessment. Sixth, planned subgroup analyses by anesthesia type, regional block use, age, or surgical category were not feasible with only eight studies and would require a larger evidence base.

Despite these caveats, the present study provides preliminary evidence that perioperative dexmedetomidine combined with esketamine may offer neuroprotective and recovery-related benefits. Large, multicenter randomized controlled trials with standardized comparator arms, adequate safety monitoring, and longer follow-up are needed to determine the optimal dosing, timing, and target populations for this combination.

## Conclusion

Based on moderate- to low-quality evidence, the perioperative combination of esketamine and dexmedetomidine may be associated with reduced neuronal injury biomarkers, improved early cognitive test scores, and lower risks of delirium, PONV, and early postoperative pain. These findings suggest a potential neuroprotective role for this combined regimen in facilitating postoperative recovery. However, the certainty of evidence for MMSE and VAS outcomes remains low, warranting cautious interpretation. Given the limited number of included studies, their single-country origin, and the absence of standardized safety reporting, the present findings should be considered preliminary. High-quality, multicenter randomized controlled trials are needed to establish optimal dosing, timing, and target populations before routine clinical adoption.

## Data Availability

The raw data supporting the conclusions of this article will be made available by the authors, without undue reservation.
