# Supplementary material for: Perioperative dexmedetomidine plus esketamine for postoperative neurocognitive outcomes: a systematic review and meta-analysis
Source: Front Med (Lausanne). 2026 Aug 20;13:1909826. doi: 10.3389/fmed.2026.1909826 (PMC13539719; doi:10.3389/fmed.2026.1909826)
Supplement: Supplementary file 1 [file Data_Sheet_1.docx]

**Supplementary Table S1.** PubMed Search Strategy

| Search Set | Search Terms |
| --- | --- |
| #1 | "Dexmedetomidine"[Mesh] OR Dexmedetomidine OR Dexmedetomidine Hydrochloride OR MPV-1440 OR Precedex OR Igalmi OR Sedadex OR Sileo OR Cepedex OR Dexdor OR Dexdomitor |
| #2 | "Ketamine"[Mesh] OR Esketamine OR Ketalar OR CI-581 OR Ketanest OR Ketamine Hydrochloride OR Calipsol OR Calypsol OR Kalipsol OR Ketaset |
| #3 | "Postoperative Cognitive Complications"[Mesh] OR postoperative cognitive dysfunction OR Cognitive Complication, Postoperative OR Postoperative Cognitive Decline OR Postoperative Cognitive Dysfunction OR Postoperative Cognitive Disorder OR Postoperative Dementia |
| #4 | Emergence Delirium OR Agitated Emergence OR Emergence Agitation OR Postoperative Delirium |
| #5 | "Cognitive Dysfunction"[Mesh] OR Cognitive Impairment OR Mild Cognitive Impairment OR Cognitive Decline OR Mental Deterioration |
| #6 | "Neurocognitive Disorders"[Mesh] OR Neurocognitive Disorder OR Mild Neurocognitive Disorder |
| Final | #1 AND #2 AND (#3 OR #4 OR #5 OR #6) |

**Note:** The search was limited to primary clinical studies. Terms such as "meta-analysis" or "systematic review" were intentionally omitted from the main search strategy to avoid retrieving secondary publications. The search was conducted from database inception to April 2026, with no language or study design restrictions applied.

**Supplementary Figure S1.** Results of the sensitivity analysis using the leave-one-out method.

The plots illustrate the stability of the pooled effect estimates for serum S-100β levels, cognitive scores (MMSE, MoCA), postoperative delirium, PONV, and pain scores. Each interval represents the 95% confidence interval of the summary estimate after excluding the study indicated on the y-axis. The consistency of these estimates supports the robustness of the meta-analysis results, suggesting limited influence of potential publication bias.

**Supplementary Figure S2.** Sensitivity analysis excluding the non-randomized study (Li_2025).

Forest plots show pooled effect estimates for major outcomes (24-h MMSE and MoCA scores, POD, and PONV) after restricting the analysis to randomized controlled trials only. Effect estimates are presented as standardized mean difference (SMD) or risk ratio (RR) with 95% confidence intervals.
